# Robot-Assisted Kyphoplasty Improves Clinical and Radiological Features Better Than Fluoroscopy-Assisted Kyphoplasty in the Treatment of Vertebral Compression Fractures: A Meta-Analysis

**DOI:** 10.3389/fsurg.2022.955966

**Published:** 2022-07-05

**Authors:** Hongwei Yu, Gan Luo, Bin Yu, Tianwei Sun, Qiong Tang, Yutao Jia

**Affiliations:** ^1^ School of Medicine, Nankai University, Tianjin, China; ^2^ Department of Spinal Surgery, Tian-jin Union Medical Centre, Nankai University People’s Hospital, Tianjin, China; ^3^ Graduate School of Tianjin Medical University, Tianjin, China; ^4^ Department of Respiratory Medicine, Tian-jin Union Medical Centre, Nankai University People’s Hospital, Tianjin, China

**Keywords:** Robotic-assisted, fluoroscopy-assisted, kyphoplasty, vertebral compression fractures, vertebral height, vertebral kyphosis angle, cement leakage rates

## Abstract

**Purpose:**

This meta-analysis aimed to determine whether patients treated with robot-assisted kyphoplasty for vertebral compression fractures have superior clinical and radiographic improvement than those treated with fluoroscopy.

**Methods:**

A comprehensive search of the PubMed, Embase, Cochrane Library, Science Direct, and CNKI (China National Knowledge Infrastructure) databases was conducted to find randomized control trials (RCTs) or observational cohort studies that compared robotic-assisted kyphoplasty (RA-kyphoplasty) with fluoroscopy-assisted kyphoplasty (FA-kyphoplasty) in treating vertebral compression fractures. Preoperative, postoperative, and final follow-up data on vertebral height (VH), vertebral kyphosis angle (VKA), visual analog scale (VAS) for back pain, and cement leakage rate were collected from eligible studies for meta-analysis. Patients were divided into RA and FA groups depending on whether the operation was robotically or fluoroscopically guided.

**Results:**

We included 6 cohort studies with 491 patients and 633 vertebrae. The results of the meta-analysis showed that the RA group had a higher VH than the FA group at both postoperation (*p* < 0.001) and final follow-up (*p* < 0.001); the VKA in the RA group was lower than that in the FA group at postoperation (*p* < 0.001) and final follow-up (*p* < 0.001); the back pain VAS score was lower in the RA group than in the FA group at postoperation (*p* = 0.01) and final follow-up (*p* = 0.03); and the cement leakage rate in the RA group was lower than those in the FA group (*p* < 0.001).

**Conclusion:**

This meta-analysis demonstrated that RA-kyphoplasty outperformed FA-kyphoplasty in vertebral height restoration, kyphosis angle correction, VAS score reduction for back pain, and lower cement leakage rate in the treatment of vertebral compression fractures.

## Introduction

Kyphoplasty is commonly applied in the treatment of OVFs, with the advantages of rapid pain relief and correction of vertebral height and kyphosis (1, 2). This is essential for improving the quality of life and maintaining the sagittal balance of the spine for patients (3, 4). However, the correction of vertebral height and kyphosis with this technique is often not thorough enough to completely correct the sagittal imbalance of the spine, so that patients have to compensate for the anterior shift of the body through lumbar extension or pelvic inversion (5). This non-physiological compensation will lead to chronic persistent low back pain (6) and increase the risk of vertebral refracture (7). In addition, both surgeons and patients may be at risk for dermatitis, cataracts, and cancer due to the high radiation exposure caused by repeated fluoroscopy (8, 9).

Robotic assistance enables three-dimensional planning of the K-wire's position, potentially allowing precise placement of vertebral implants. Advantages of the robot over fluoroscopic techniques also include prevention of tremors, avoidance of instability caused by manual manipulation, and further reduction of damage to the pedicle and posterior wall of the vertebral body, thereby reducing the risk of cement leakage (10, 11).

This meta-analysis aimed to evaluate whether patients treated with robot-assisted kyphoplasty for vertebral compression fractures have superior clinical and radiographic improvement than patients treated with fluoroscopy-assisted kyphoplasty.

## Methods

The Preferred Reporting Items for Systematic Reviews and Meta-Analyses (PRISMA) statement (12) was used as guidance for our systematic review and meta-analysis (see Supplementary file for PRISMA checklist).The protocol for this review was registered on the International Platform of Registered Systematic Review and Meta-analysis Protocols database with the registration number INPLASY202250106 and DOI number 10.37766/inplasy2022.5.0106.

### Search Strategy and Study Selection

The following databases were extensively searched: PubMed, EMBASE, ScienceDirect, the Cochrane Library, and CNKI. We identified relevant articles published up to 1 May 2022 without language limitations. Studies were found using the following keywords: “spinal robot”, “robot-assisted”, “vertebral compression fracture”, and “kyphoplasty”. Two independent investigators screened eligible studies and reviewed references of included studies to identify additional articles. A third reviewer was consulted when the two reviewers could not reach a consensus.

### Selection Strategy

The inclusion and exclusion criteria of studies followed PICOS principles. (1) Participants: Patients with osteoporotic vertebral compression fractures or traumatic vertebral compression fractures (Magerl type A), who need to be treated with kyphoplasty. (2) Interventions: Kyphoplasty was performed under robotic guidance. (3) Comparisons: Kyphoplasty was performed under conventional fluoroscopic guidance (4) Outcomes: studies should include at least one of the following data: preoperative, postoperative and final follow-up vertebral height and kyphosis angle and VAS score for back pain; cement leakage rate. (5) Study design: Observational studies and randomized control trials were eligible. Case reports, case series, commentaries, practice guidelines, systematic reviews and meta analysis were excluded. In addition, duplicate studies with the same cohort or studies considered by consensus to be of low quality were excluded.

### Data Extraction

Data were extracted from the included studies as follows: (1) study design: first author, publication region, publication time, and study type; (2) sample demographics: number of patients and vertebrae, follow-up time, age, sex, and disease diagnosis; (3) surgery details: robot type, operation time, X-ray exposure frequency and doses; and (4) analysis variables: preoperative, postoperative and final follow-up vertebral height and kyphosis angle and VAS score for back pain and cement leakage rate. The results could not be meta-analyzed for the operation time, radiation exposure frequency, and doses due to the significant heterogeneity among the reported outcomes across all studies.

### Assessment of Risk of Bias

Two reviewers evaluated bias risk in the cohort studies using the Newcastle–Ottawa scale (13). Sensitivity analysis was performed by excluding a single study of each study in turn and reanalyzing the data. Publication bias was analyzed qualitatively by funnel plot.

### Statistical Analysis

The continuous variables were estimated by weighted mean difference (WMD), and dichotomous variables were estimated by using odds ratios (ORs) with 95% confidence intervals (CIs). The statistical heterogeneity of the pooled results was determined using the *I*^2^ statistic. For this meta-analysis, we used the fixed-effect model when *I*^2^ was greater than 50%, and if *I*^2^ was less than 50%, a random-effect model was applied. The meta-analysis results were considered statistically significant when the *p* value was <0.05. The meta-analysis was performed using Review Manager 5.4 (Revman, The Cochrane Collaboration, Oxford, The UK).

## Results

### Search Results

**Figure 1:**
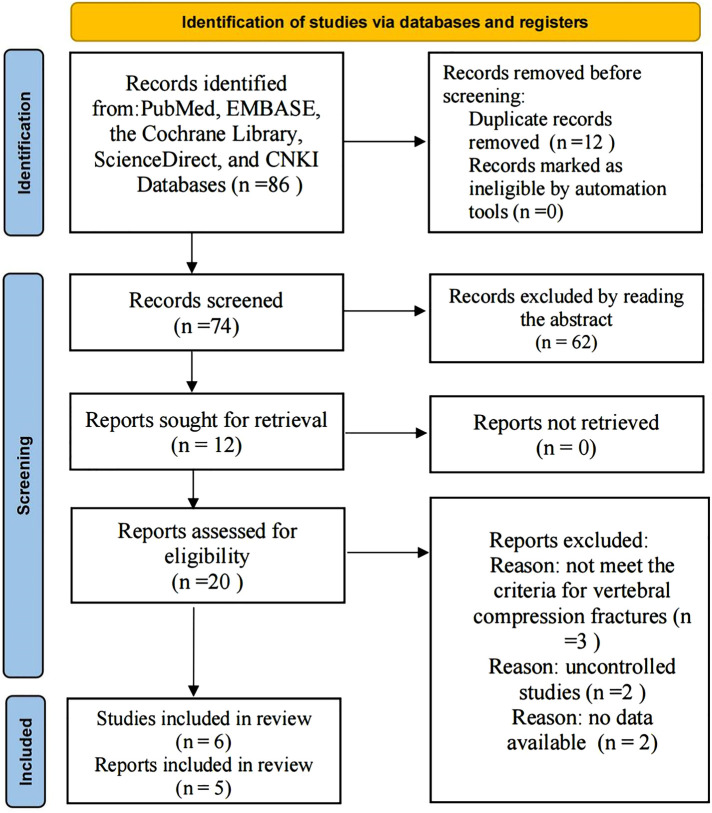
PRISMA flowchart.

A total of 86 articles from PubMed, EMBASE, the Cochrane Library, ScienceDirect, and CNKI were initially identified. The exact number of articles identified in each database is as follows: PubMed (*n* = 65), EMBASE (*n* = 17), ScienceDirect (*n* = 3), Cochrane library (*n* = 0), CNKI (*n* = 1). Twelve articles were excluded because of duplication, and 62 articles were excluded by screening the titles and abstracts individually. There remained 12 articles that underwent a comprehensive full-text analysis. Finally, 5 articles (14–18) met the inclusion and exclusion criteria. Because the article by Yuan (18) contains two cohort studies, a single-segment cohort (S) and a double-segment cohort (D), 6 studies were included in the final meta-analysis. The flow chart used for the new systematic review according to PRISMA 2020 is shown in Figure 1.

### Study Characteristics and Risk of Bias

A total of 491 patients and 633 vertebrae were involved in the 6 studies. The RA group included 232 patients and 311 vertebrae, and the FA group included 259 patients and 322 vertebrae. The characteristics of the included studies are presented in Table 1. All 6 cohort studies were evaluated by the Newcastle–Ottawa scale (NOS). The evaluation results are shown in Table 2.

**Table 1:** Demographics and characteristics of included studies.

Author	Year	Country	Study Design	RA/FA					
				Patients Size	Vertebrae size	Age (mean, year)	Diagnosis	Robot types	Mean Follow-Up (mos)
Alsalmi	2019	France	cohort studies	30/30	35/32	49.4/40.6	TVCF	ROSA	/
Jin	2021	China	cohort studies	81/131	81/131	75.44/72.65	OVCF	TiRobot	6.87/7.04
Lin	2020	China	cohort studies	33/30	86/75	68.8/67.9	OVCF	TiRobot	11/13
Wang	2021	China	cohort studies	30/30	30/30	66/76	OVCF	TiRobot	/
Yuan (d)	2020	China	cohort studies	37/22	37/22	71.5/70.3	OVCF	TiRobot	6
Yuan (s)	2020	China	cohort studies	21/16	42/32	69.9/73.4	OVCF	TiRobot	6

*TVCF: traumatic vertebral compression fracture; OVCF: osteoporotic vetebral compression fracture.*

**Table 2:** Newcastle-Ottawa scale for observational studies.

Author	Year	Selection	Comparability	Outcomes	Quality judgment
Alsalmi	2019	4	2	3	9
Jin	2021	4	1	2	7
Lin	2020	4	2	2	8
Wang	2021	4	1	2	7
Yuan (d)	2020	4	2	2	8
Yuan (s)	2020	4	2	2	8

*Selection: (1) representativeness of the exposed cohort, (2) selection of the nonexposed cohort, (3) ascer tainment of exposure and (4) demonstration that outcome of interest was not present at the start of stud.*

*Comparability: comparability of cohorts on the basis of the design or analysis.*

*Outcomes: (1) assessment outcome, (2) was follow-up long enough for outcomes to occur, (3) adequacy of follow-up of cohorts (≥1 years)*.

*NOS scores ≥ 7 indicate a high-quality study.*

### Meta-Analysis Results

The summary of the results of RA-kyphoplasty versus FA-kyphoplasty are shown in Table 3.

**Table 3:** Summarized results of robotic-assisted kyphoplasty versus fluoroscopy-assisted kyphoplasty.

Variables	No. of studies	Heterogeneity test		Effect size			Model	Conclusion
		*I*^2^ (%)	*p* value	OR/WMD	95%CI	*p* value		
VH								
Postoperative VH	3	0	0.37	2.89	1.72/4.06	*p* < 0.001	Fixed	Signifcant
Final follow-up VH	3	0	0.99	2.94	1.93/3.94	*p* < 0.001	Fixed	Signifcant
VKA								
Postoperative VKA	6	65	0.01	−1.99	−2.93/−1.05	*p* < 0.001	Random	Signifcant
Final follow-up VKA	4	54	0.09	−2.19	−3.27/−1.10	*p* < 0.001	Random	Signifcant
VAS score for back pain								
Postoperative VAS	6	0	0.66	−0.23	−0.41/−0.06	0.01	Fixed	Signifcant
Final follow-up VAS	4	0	0.95	−0.14	−0.28/−0.01	0.03	Fixed	Signifcant
cement leakage	6	0	0.80	0.29	0.17/0.49	*p* < 0.001	Fixed	Signifcant

*VH, vetebral height; VKA, vetebral kyphosis angle; VAS, visual analogue scale.*

#### Vertebral Height

**Figure 2:**
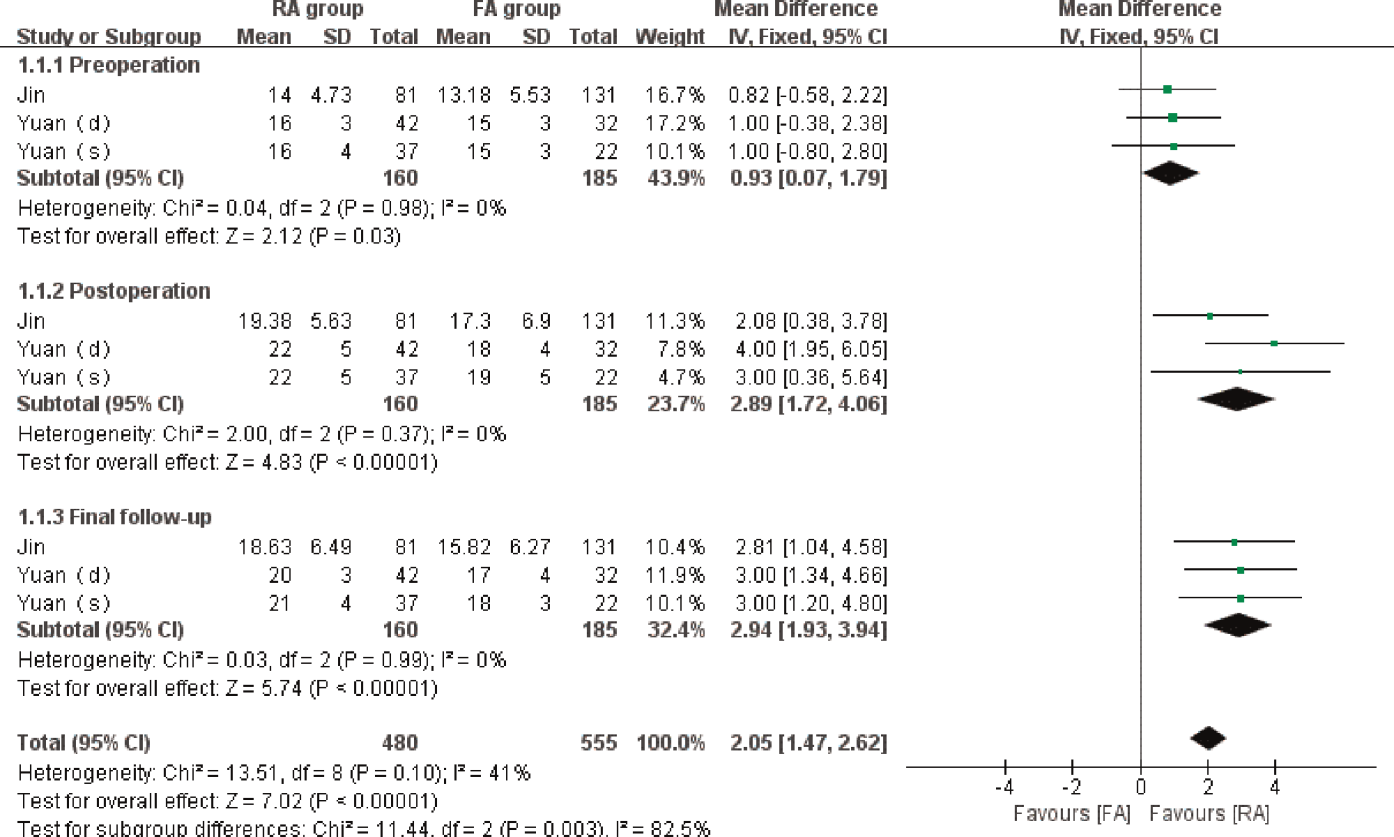
Forest plot depicting WMDs of the vertebral height.

A total of 3 studies (15, 18) reported the preoperative, postoperative, and final follow-up vertebral height in 345 vertebrae (160 vertebrae in the RA group and 185 vertebrae in the FA group). No significant statistical heterogeneity was detected(*I*^2 ^= 0% in each subgroup). The outcomes indicated that although the degree of preoperative vertebral body compression in the RA group was slightly lower than that in the FA group, the restored height of the vertebrae tended to be significantly greater in the RA group than that in the FA group following the operation. Slightly statistically difference in the preoperative vertebral height (WMD = 0.93, 95% CI:(0.007,1.79), *p* = 0.03), significant statistically differences in vertebral height were observed at postoperation (WMD = 2.89, 95% CI:(1.72,4.06), *p* < 0.001) and final follow-up (WMD = 2.94, 95% CI:(1.93, 3.94), *p* < 0.001). (Figure 2).

#### Vertebral Kyphosis Angle

**Figure 3:**
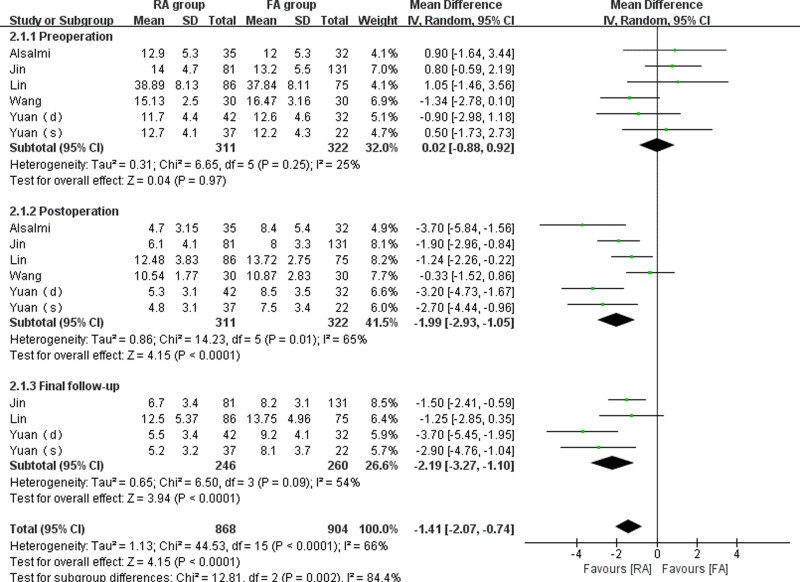
Forest plot depicting WMDs of the vertebral kyphosis angle.

A total of 6 studies (14–18) reported preoperative, postoperative, and final follow-up kyphosis angles in 633 vertebrae (311 vertebrae in the RA group and 322 vertebrae in the FA group). Because minor statistical heterogeneity was detected (*I*^2 ^= 25%, 65%, 54%, respectively), the random-effect model was used to analyze the merged variables. The outcomes indicated that the preoperative vertebral kyphosis angle was not significantly different between the groups (WMD = 0.02, 95% CI: (−0.88, 0.92), *p* = 0.97), while the kyphosis angles were significantly different between the groups postoperatively (WMD = −1.99, 95% CI: (−2.93, −1.05), *p* < 0.001) and at the final follow-up (WMD = −2.19, 95% CI: (−3.27, −1.10), *p* < 0.001) (Figure 3). According to the findings, robot-assisted kyphoplasty is more effective for correcting kyphotic deformities caused by vertebral compression.

#### VAS Score for Back Pain

**Figure 4:**
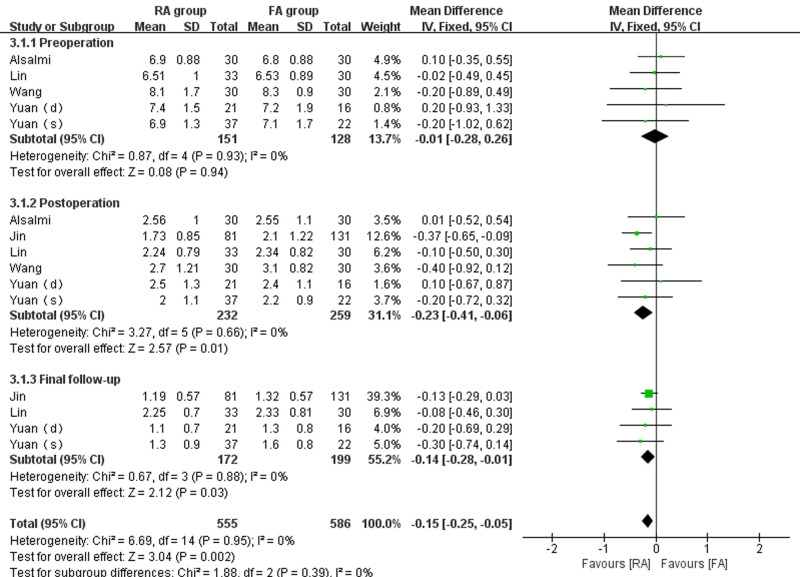
Forest plot depicting WMDs of the VAS scores for back pain.

Six studies (14–18) compared the preoperative, postoperative, and final follow-up VAS scores of the RA and FA groups (232 patients in the RA group and 259 patients in the FA group). No significant statistical heterogeneity was found (*I*^2 ^= 0% in each subgroup). There was no statistically detectable difference between the RA group and the FA group in preoperative VAS score (WMD = −0.01, 95% CI: (−0.28, 0.26), *p* = 0.94). However, there was a statistically significant difference between patients undergoing RA-kyphoplasty versus those undergoing FA-kyphoplasty regarding postoperative back pain VAS score (WMD = −0.23, 95% CI:(−0.41, 0.06), *p* = 0.01) and final follow-up back pain VAS score (WMD = −0.14, 95% CI:(−0.28, 0.01), *p* = 0.03) (Figure 4).

#### Cement Leakage Rate

**Figure 5:**
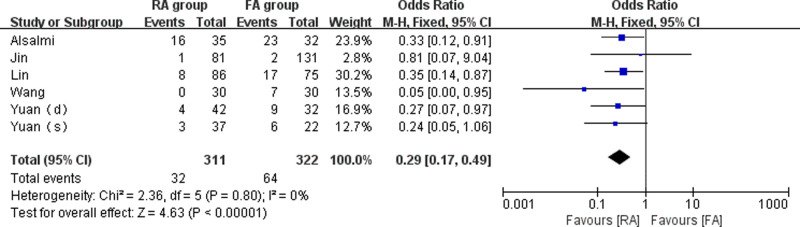
Forest plot depicting the OR of the cement leakage rates.

Six studies (14–18) compared the cement leakage rate between the RA group and the FA group (311 vertebrae in the RA group and 322 vertebrae in the FA group). The meta-analysis was conducted using a fixed-effect model with *I*^2^ = 0%. The outcomes indicated that cement leakage rate was lower in the RA group than in the FA group (OR  = 0.29, 95% CI: (0.17, 0.49), *p* < 0.001). (Figure 5).

#### Sensitivity Analyses and Publication Bias

**Figure 6:**
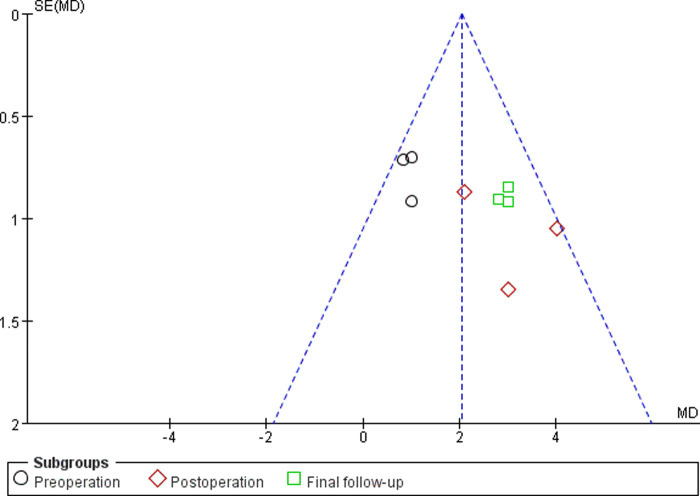
Funnel plot of vertebral height.

**Figure 7:**
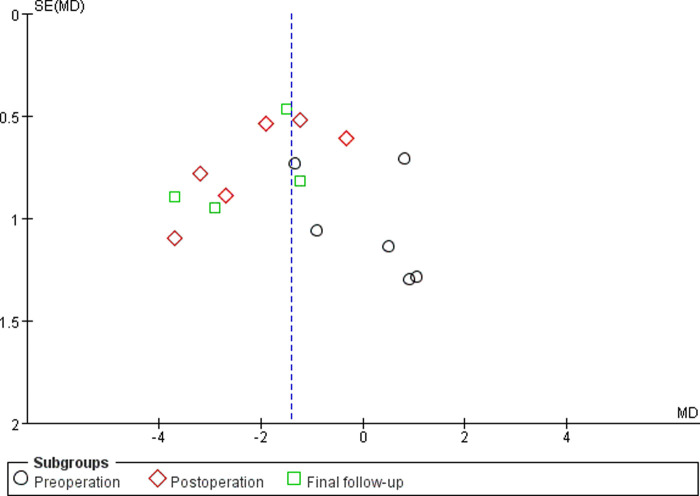
Funnel plot of the vertebral kyphosis angle.

**Figure 8:**
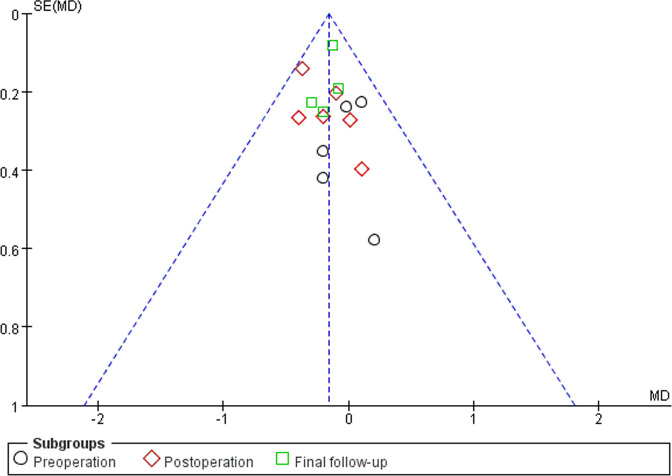
Funnel plot of VAS scores for back pain.

**Figure 9:**
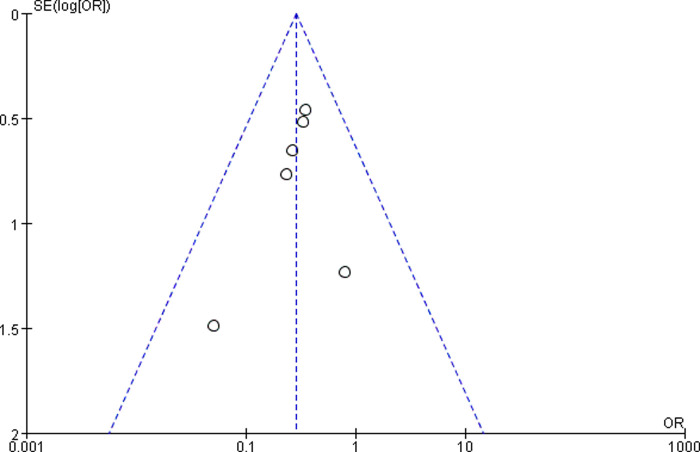
Funnel plot of cement leakage rates.

Sensitivity analyses did not provide different results in terms of vertebral height, vertebral kyphosis angle, VAS score for back pain and cement leakage rate, by reanalyzing the data after sequential single elimination of each studies. Publication bias was not evident based on funnel plots as shown in Figures 6–9.

## Discussion

This meta-analysis investigated the effects of robot use on postoperative clinical and radiological outcomes for patients undergoing kyphoplasty surgery. The results showed that RA-kyphoplasty produced better results than FA-kyphoplasty with regard to vertebral height, vertebral kyphosis angle, VAS score for back pain, and cement leakage rates postoperatively and also at the final follow-up.

Correction of vertebral height and kyphosis angle appears to be important factors in a patient's overall improvement. It is necessary to reestablish the sagittal balance of the spine after vertebral compression fractures, which may be particularly problematic in younger patients (19, 20). However, the correction of the sagittal balance of the spine is often inadequate, as the range of motion of a fluoroscopy-guided needle is often limited, especially when accompanied by scoliosis or slipped deformity (21), because fluoroscopy-assisted pedicle puncture relies on anatomical landmarks for positioning. In addition, for the upper thoracic vertebrae (T1–T4), the pedicles are small and obstructed by the humeral head and scapula, making it difficult to identify clearly by intraoperative fluoroscopy. Therefore, one of the limitations of fluoroscopic pedicle puncture is that the balloon or other implant cannot be placed in the most severely collapsed vertebral body. Robot-assisted technology can theoretically solve the above problems and is valuable for the optimal positioning of the implants. The entry point, orientation, and final position of the implant can be planned preoperatively. The position of the implants in the vertebral body can be continuously optimized through 3D visualization. This function allows the implants to be placed precisely according to the surgeon’s plan. For example, the implant can be placed at the point of the lowest collapse in the vertebral body, which not only allows for better restoration of vertebral height but also reduces damage to the wall of the vertebral body and pedicle during the implantation process. In addition, the robot can eliminate the errors caused by surgeon's fatigue or tremors during manual operation, which also helps obtain the optimal position of the implants.

Several previous studies (22–24) have shown that the distribution of bone cement is related to residual pain after kyphoplasty. With RA-kyphoplasty, the implants can be accurately positioned near the midline or fracture line, allowing the cement to be diffusely distributed along the midline of the vertebral body or fractured area, which can provide better pain relief. In addition, traditional fluoroscopy-assisted pedicle puncture often requires repeated adjustment of the puncture angle to ensure that the puncture needle does not violate the wall of the pedicle. This will inevitably aggravate the damage to muscles, fascia, and other soft tissues, resulting in inadequate postoperative pain relief. In contrast, RA-kyphoplasty is free of these problems. Our meta-analysis showed that the RA group experienced more adequate pain relief than the FA group, and this advantage persisted at the final follow-up, which may be related to the more adequate correction of vertebral kyphosis in the robot group (25, 26).

Cement leakage after kyphoplasty is a problem to be solved, and specific morbidity has been reported in the literature (27). Our meta-analysis showed a marked difference in cement leakage rate between the RA group and the FA group (10.3% vs. 19.9%, *p* < 0.001). Undoubtedly, the precision of the robot-assisted puncture can prevent damages to the wall of the pedicle and vertebral body, thereby reducing the risk of cement leakage. However, cement leakage can still be caused by a variety of factors, such as the amount of cement injected, fracture severity, and intravertebral fissures (28, 29). Additional reasons for the reduction of cement leakage with RA-kyphoplasty need to be further explored in randomized controlled trials with larger sample sizes.

Frequency and doses of radiation exposure and operative time are important parameters for evaluating the merits of robot-assisted surgery. Unfortunately, those data could not be pooled for analysis because of the large heterogeneity among the included studies. The differences in frequency and doses of radiation exposure were due to differences in the registration design of different robots. The “Mazor” (“SpineAssist”, “Renaissance”) robotic system plans the puncture path through three-dimensional images from computed tomography (CT) before surgery and performs the registration procedure only through the anterior-posterior and lateral position images from the C-arm during surgery. However, the “Rosa” and “TiRobot” robotic systems require automatic registration through intraoperative three-dimensional C-arm scanning. Although intraoperative three-dimensional C-arm scanning significantly increases the patient’s radiation exposure doses during surgery, it can reduce the total radiation exposure doses during hospitalization because three-dimensional C-arm scanning at the end of surgery can replace routine postoperative CT scanning (14). We favor this approach because the radiation doses from CT scanning are much greater than those from intraoperative three-dimensional C-arm scanning. The operation time varies depending on the type of robot, proficiency level of the operator, and location and number of fractured segments. Critics believe that RA-kyphoplasty is complex and time-consuming; for example, Yuan (18) used the “TiRobot” robot to perform single-segment kyphoplasty, which required 45.4 ± 6.1 min, and this was higher than that of the FA-kyphoplasty (36.1 ± 5.7 min). He believed that the main reason for the longer operation duration was the additional preparation time required by the robot. Jin (15) also used the “TiRobot” robot to perform single-segment kyphoplasty, and the procedure time was 37.33 ± 4.81 min, which was shorter than that of the FA-kyphoplasty (47.21 ± 6.10 min). We believe that the pre-surgical preparation of the robot could be carried out by a skilled radiographer, and surgeons work methodically with other operating room staff so that the preparation of the robot does not become a waste of time.

Most clinical studies focus on the accuracy of robotic-assisted pedicle screw fixation in spine surgery (30, 31). However, the potential value of robot-assisted kyphoplasty in improving the reliability and quality of vertebral height restoration has not been evaluated. The results of this meta-analysis may play a role in the exploration of spinal robotics from assisting pedicle screw placement to assisting different surgical approaches for various types of spinal disorders, such as robot-assisted kyphoplasty.

### Limitations

First, there were no randomized controlled trials in any of the included studies. However, funnel plots indicated minimal to no bias for observational studies, as shown in Figures. 6–9. Second, although all samples were patients with vertebral compression fractures, different causes of fractures may potentially affect outcomes. Third, radiation exposure frequency and doses and operation time could not be pooled for analysis due to the substantial heterogeneity between the included studies. Fourth, randomized controlled trials or observational studies with longer follow-ups are needed to supplement the existing conclusions.

## Conclusions

This meta-analysis demonstrated that RA-kyphoplasty outperformed FA-kyphoplasty concerning vertebral height restoration, kyphosis angle correction, VAS score reduction for back pain, and reduction of cement leakage in the treatment of vertebral compression fractures.

## Data Availability

The original contributions presented in the study are included in the article/Suplementary Material, further inquiries can be directed to the corresponding author/s.

## References

[1] Yang H Liu H Wang S Wu K Meng B Liu T. Review of percutaneous kyphoplasty in China. Spine. (2016) 41(Suppl 19):b52–b8. 10.1097/BRS.0000000000001804 27656784

[2] Hartmann F Gercek E Leiner L Rommens PM. Kyphoplasty as an alternative treatment of traumatic thoracolumbar burst fractures magerl type A3. Injury. (2012) 43(4):409–15. 10.1016/j.injury.2010.03.025 20417512

[3] Chenin L Capel C Fichten A Peltier J Lefranc M. Evaluation of screw placement accuracy in circumferential lumbar arthrodesis using robotic assistance and intraoperative flat-panel computed tomography. World Neurosurg. (2017) 105:86–94. 10.1016/j.wneu.2017.05.118 28576710

[4] Hiwatashi A Westesson PL Yoshiura T Noguchi T Togao O Yamashita K Kyphoplasty and vertebroplasty produce the same degree of height restoration. AJNR Am J Neuroradiol. (2009) 30(4):669–73. 10.3174/ajnr.A1442 19131409 PMC7051743

[5] Lin T Lu J Zhang Y Wang Z Chen G Gu Y Does spinal sagittal imbalance lead to future vertebral compression fractures in osteoporosis patients? Spine J. (2021) 21(8):1362–75. 10.1016/j.spinee.2021.03.014 33766788

[6] Yang JS Liu JJ Chu L Li J Chen C Chen H Causes of residual back pain at early stage after percutaneous vertebroplasty: a retrospective analysis of 1,316 cases. Pain Physician. (2019) 22(5):e495–e503. 10.36076/ppj/2019.22.e495 31561662

[7] Ji C Rong Y Wang J Yu S Yin G Fan J Risk factors for refracture following primary osteoporotic vertebral compression fractures. Pain Physician. (2021) 24(3):e335–e40. 10.36076/ppj.2021/24/e335 33988955

[8] Loisel F Menu G Boyer E Pluvy I Obert L. Radiation exposure and the orthopedic surgeon's hand: measurement of the equivalent dose over 13 months. Hand Surg Rehabil. (2017) 36(2):97–101. 10.1016/j.hansur.2016.11.006 28325434

[9] Mroz TE Abdullah KG Steinmetz MP Klineberg EO Lieberman IH. Radiation exposure to the surgeon during percutaneous pedicle screw placement. J Spinal Disord Tech. (2011) 24(4):264–7. 10.1097/BSD.0b013e3181eed618 20844448

[10] Barzilay Y Schroeder JE Hiller N Singer G Hasharoni A Safran O Robot-assisted vertebral body augmentation: a radiation reduction tool. Spine. (2014) 39(2):153–7. 10.1097/BRS.0000000000000100 24173014

[11] Yuan W Cao W Meng X Zhu H Liu X Cui C Learning curve of robot-assisted percutaneous kyphoplasty for osteoporotic vertebral compression fractures. World Neurosurg. (2020) 138:e323–e9. 10.1016/j.wneu.2020.02.110 32112940

[12] Parums DV. Editorial: review articles, systematic reviews, meta-analysis, and the updated preferred reporting items for systematic reviews and meta-analyses (PRISMA) 2020 guidelines. Med Sci Monit. (2021) 27:e934475. 10.12659/msm.934475 34421116 PMC8394590

[13] Stang A. Critical evaluation of the Newcastle-Ottawa scale for the assessment of the quality of nonrandomized studies in meta-analyses. Eur J Epidemiol. (2010) 25(9):603–5. 10.1007/s10654-010-9491-z 20652370

[14] Alsalmi S Capel C Chenin L Peltier J Lefranc M. Robot-assisted intravertebral augmentation corrects local kyphosis more effectively than a conventional fluoroscopy-guided technique. J Neurosurg Spine. (2018) 30(2):289–95. 10.3171/2018.8.SPINE18197 30544363

[15] Jin M Ge M Lei L Li F Wu M Zhang G Clinical and radiologic outcomes of robot-assisted kyphoplasty versus fluoroscopy-assisted kyphoplasty in the treatment of osteoporotic vertebral compression fractures: a retrospective comparative study. World Neurosurg. (2021) S1878-8750(21)01563-1. 10.1016/j.wneu.2021.10.066 34637939

[16] Lin S Hu J Wan L Tang L Wang Y Yu Y [Robot-guided percutaneous kyphoplasty in treatment of multi-segmental osteoporotic vertebral compression fracture]. Zhongguo Xiu Fu Chong Jian Wai Ke Za Zhi. (2020) 34(9):1136–41. 10.7507/1002-1892.202002131 32929907 PMC8171738

[17] Wang B Cao J Chang J Yin G Cai W Li Q Effectiveness of tirobot-assisted vertebroplasty in treating thoracolumbar osteoporotic compression fracture. J Orthop Surg Res. (2021) 16(1):65. 10.1186/s13018-021-02211-0 33468187 PMC7816462

[18] Yuan W Meng X Cao W Zhu Y. Robot-Assisted versus fluoroscopy-assisted kyphoplasty in the treatment of osteoporotic vertebral compression fracture: a retrospective study. Global Spine J. (2022) 12(6):1151–7. 10.1177/2192568220978228 33375861 PMC9210249

[19] Chau LTC Hu Z Ko KSY Man GCW Yeung KH Law YY Global sagittal alignment of the spine, pelvis, lower limb after vertebral compression fracture and its effect on quality of life. BMC Musculoskelet. Disord. (2021) 22(1):476. 10.1186/s12891-021-04311-8 34030686 PMC8146251

[20] McLain RF. Functional outcomes after surgery for spinal fractures: return to work and activity. Spine. (2004) 29(4):470–7; discussion Z6. 10.1097/01.BRS.0000092373.57039.FC 15094545

[21] Pang J Liu B Chen H Zhang W Sun J Zhang X. Precise puncture combined with simplified percutaneous vertebroplasty to treat osteoporotic vertebral compression fractures: a comparative analysis with conventional percutaneous vertebroplasty. Am J Transl Res. (2021) 13(12):14195–202. 10.21203/rs.3.rs-101497/v1 35035765 PMC8748117

[22] Li Y Yue J Huang M Lin J Huang C Chen J Risk factors for postoperative residual back pain after percutaneous kyphoplasty for osteoporotic vertebral compression fractures. Eur Spine J. (2020) 29(10):2568–75. 10.1007/s00586-020-06493-6 32507918

[23] Li Q Shi L Wang Y Guan T Jiang X Guo D A nomogram for predicting the residual back pain after percutaneous vertebroplasty for osteoporotic vertebral compression fractures. Pain Res Manag. (2021) 2021:3624614. 10.1155/2021/3624614 34760032 PMC8575618

[24] Liu Z Zhang X Liu H Wang D. A nomogram for short-term recurrent pain after percutaneous vertebroplasty for osteoporotic vertebral compression fractures. Osteoporos Int. (2022) 33(4):851–60. 10.1007/s00198-021-06232-7 34762140

[25] Hoh BL Rabinov JD Pryor JC Hirsch JA. Balloon kyphoplasty for vertebral compression fracture using a unilateral balloon tamp via a uni-pedicular approach: technical note. Pain Physician. (2004) 7(1):111–4. 10.36076/ppj.2004/7/111 16868622

[26] Zhang B Li T Wang Z. Efficacy and complications of different surgical modalities of treating osteoporotic spinal compression fracture in the elderly. Am J Transl Res. (2022) 14(1):364–72. PMID: 35173854 PMC8829605

[27] Rotter R Schmitt L Gierer P Schmitz KP Noriega D Mittlmeier T Minimum cement volume required in vertebral body augmentation–A biomechanical study comparing the permanent SpineJack device and balloon kyphoplasty in traumatic fracture. Clin Biomech (Bristol, Avon). (2015) 30(7):720–5. 10.1016/j.clinbiomech.2015.04.015 25971847

[28] Zhang K She J Zhu Y Wang W Li E Ma D. Risk factors of postoperative bone cement leakage on osteoporotic vertebral compression fracture: a retrospective study. J Orthop Surg Res. (2021) 16(1):183. 10.1186/s13018-021-02337-1 33691731 PMC7945340

[29] Zhang S Wang GJ Wang Q Yang J Xu S Yang CH. A mysterious risk factor for bone cement leakage into the spinal canal through the batson vein during percutaneous kyphoplasty: a case control study. BMC Musculoskelet Disord. (2019) 20(1):423. 10.1186/s12891-019-2807-6 31510985 PMC6739913

[30] Kim HJ Jung WI Chang BS Lee CK Kang KT Yeom JS. A prospective, randomized, controlled trial of robot-assisted vs freehand pedicle screw fixation in spine surgery. Int J Med Robot. (2017) 13(3):10.1002/rcs.1779. 10.1002/rcs.1779 27672000

[31] Fan M Liu Y He D Han X Zhao J Duan F Improved accuracy of cervical spinal surgery with robot-assisted screw insertion: a prospective, randomized, controlled study. Spine. (2020) 45(5):285–91. 10.1097/BRS.0000000000003258 31568094

